# Unusual Association of Partial Fanconi Syndrome and Tumor-Induced Osteomalacia Revealed by Multiple Vertebral Fractures

**DOI:** 10.1007/s00223-025-01344-2

**Published:** 2025-01-31

**Authors:** Anne-Cécile Debrach, Matteo Coen, Sophie De Seigneux, Essia Saiji, Sana Boudabbous, Jean-Pierre Willi, Jacques Serratrice, Stéphane Genevay, Emmanuel Biver

**Affiliations:** 1 Division of Bone Diseases, Department of Medicine, Geneva University Hospitals, Geneva, Switzerland; 2 Division of General Internal Medicine, Department of Medicine, University Hospitals, Geneva, Switzerland; 3 Unit of Development and Research in Medical Education, Faculty of Medicine, University of Geneva, Geneva, Switzerland; 4 Division of Nephrology and Hypertension, Department of Medicine, Geneva University Hospitals, Geneva, Switzerland; 5 Division of Pathology, Geneva University Hospitals, Geneva, Switzerland; 6 Division of Radiology, Department of Diagnosis, Geneva University Hospitals, Geneva, Switzerland; 7 Division of Nuclear Medicine, Geneva University Hospitals, Geneva, Switzerland; 8 Division of Rheumatology, Department of Medicine, Geneva University Hospitals, Geneva, Switzerland

**Keywords:** Tumor-induced osteomalacia, hypophosphatemia, Fanconi syndrome, Fractures

## Abstract

Tumor-induced osteomalacia (TIO) is a rare acquired paraneoplastic syndrome caused by a mesenchymal tumor secreting a phosphaturic hormone called FGF23. Patients present with bone pain, fragility fractures and muscle weakness. Biochemical results show hypophosphatemia, raised serum alkaline phosphatase and reduced calcitriol. We report the case of a 44-year-old man who presented to the Emergency Departement with acute low back pain revealing extensive subchondral fractures between D2 and L5. Investigations showed partial Fanconi syndrome; nevertheless, he had profound hypophosphatemia, low 1,25-OH vitamin D and raised FGF23 levels suggesting a diagnosis of tumor-induced osteomalacia. A subcutaneous lesion was identified in the left leg on a PET-CT initially performed to rule out malignancy in the context of Fanconi syndrome. Tumorectomy enabled complete resolution of the electrolyte disturbances within days of surgery. This case shows that TIO may present as partial Fanconi syndrome, highlighting the importance of testing other electrolytes in cases of hypophosphatemia and the need to look for TIO in cases of partial Fanconi with severe hypophosphatemia.

## Introduction

Tumor-induced osteomalacia (TIO), also known as oncogenic osteomalacia, is a rare paraneoplastic syndrome caused by tumors secreting fibroblast growth factor-23 (FGF23), a hormone inducing renal phosphate wasting and subsequent hypophosphatemia. Patients with TIO may initially be asymptomatic or present with fatigue, weakness, generalized bone pain or fractures [1]. Fanconi syndrome (FS) is a defect in the proximal tubule of the kidney, inherited or acquired, leading to electrolyte disturbances [2]. We describe an unusual case of a young patient who presented with multiple spontaneous vertebral fractures due TIO associated with a partial FS.

## Case Report

A 44-year-old Caucasian man presented to the Emergency Department with acute mechanical lower back pain, which occurred suddenly as he rotated his trunk while sitting. The patient denied any neurological, cauda equina or B symptoms (night sweats, fever or unintentional weight loss). His past medical history and family history were unremarkable.

**Fig. 1:**
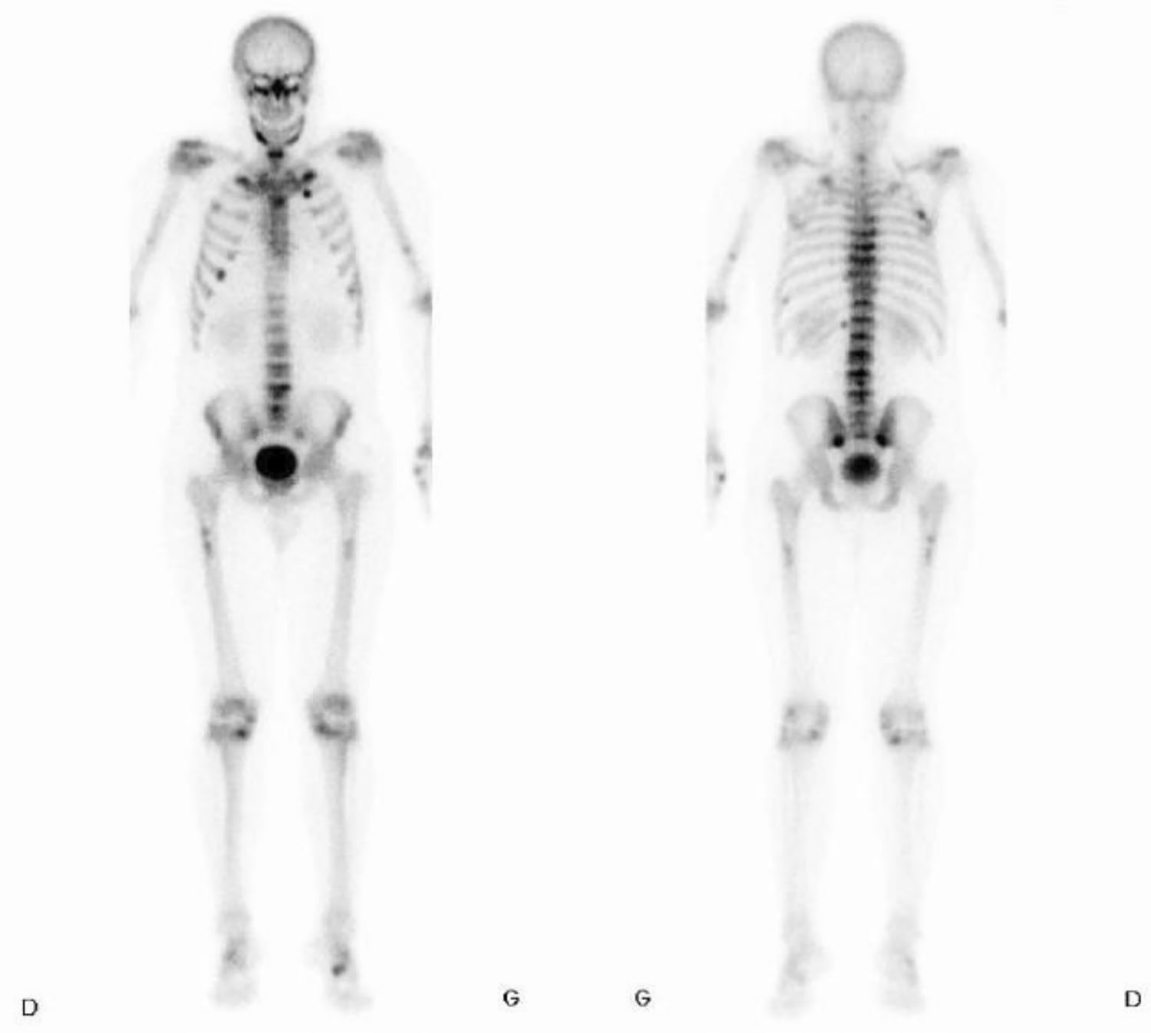
Bone scan showing diffuse bone hypefixation with several hyperfixating foci corresponding to non-consolidated fractures of the ribs, pelvis and vertebrae

Apart from tenderness on percussion of the L4 and L5 vertebrae and the presence of a paravertebral muscle contracture, physical examination was unremarkable. Lumbar spine radiographs were reported as normal, but an MRI of the spine identified acute subchondral fractures of all the vertebrae from D2 to L5. Dual-Energy X-ray Absorptiometry (DEXA) scan found normal values for age at the hip, with Z-scores of − 1.2 SD at the femoral neck and − 1.0 DS at the total hip, but a low Z-score of − 2.8 SD at the lumbar spine, with an osteoporotic T-score value of − 3.0 SD at this site, though not really interpretable due to the lumbar fractures. A bone scan highlighted diffuse hyperfixation foci corresponding to non-consolidated fractures of the ribs, the sacrum and all the dorsal and lumbar vertebrae (Fig. 1).

Blood tests showed severe hypophosphatemia (0.33 mmol/l; normal range (NR): 0.8–1.45), mild hypocalcemia with corrected calcium levels at 2.18 mmol/l (NR: 2.20–2.50 mmol/l), raised alkaline phosphatase (209 U/l; NR: 25–102), slightly reduced 25-OH vitamin D (47 nmol/l; NR: 75–105) and 1.25-OH vitamin D (33 pmol/l; NR:40–140). Parathyroid hormone (PTH) levels were mildly elevated (7.10 pmol/l, NR: 1.1–6.8). Hypokalemia (3.4 mmol/l; NR: 3.6–4.6) and hypouricemia (235 µmol/l; NR: 286–518) were also present. Creatinine (72 μmol/l, NR: 62–106), arterial blood pH (7.43) and bicarbonate levels (24.5 mmol/l; NR: 22–27) were normal. Bone turnover markers were elevated with β-crosslaps and amino-terminal propeptide of type I collagen (P1NP), respectively, at 736 ng/l (NR: 158–442) and 69 μg/l (NR: 15.1–58.6). TSH, tryptase, anti-tissue transglutaminase, immunglobulin-A antibody and β_2_-microglobulin values were normal; HIV serology was negative. Serum electrophoresis and immunofixation did not detect any abnormal proteins, and levels of urinary free light chains were normal.

Urinalysis revealed microalbuminuria and normoglycemic glycosuria (83.2 mmol/l, normal (N) < 15). Tubular maximal phosphate reabsorption/glomerular rate (TmP/GFR) was very low at 0.27 mmol/GFR (NR: 0.80–1.35), consistent with renal phosphate wasting. Urinary glycine levels were also elevated (497 mmol/mol; N < 280).

Calcium and potassium levels normalized after oral supplementation, but phosphate levels remained low despite oral and intravenous administration, combined with calcitriol substitution.

Hypokalaemia, hypouricemia, glycosuria, microalbuminuria and aminoaciduria, together with tubular phosphate wasting, without renal failure or metabolic acidosis, suggested partial Fanconi syndrome. Drug history did not identify any culprit medication. The levels of heavy metals (cadmium, mercury and lead) came back as normal.

**Fig. 2:**
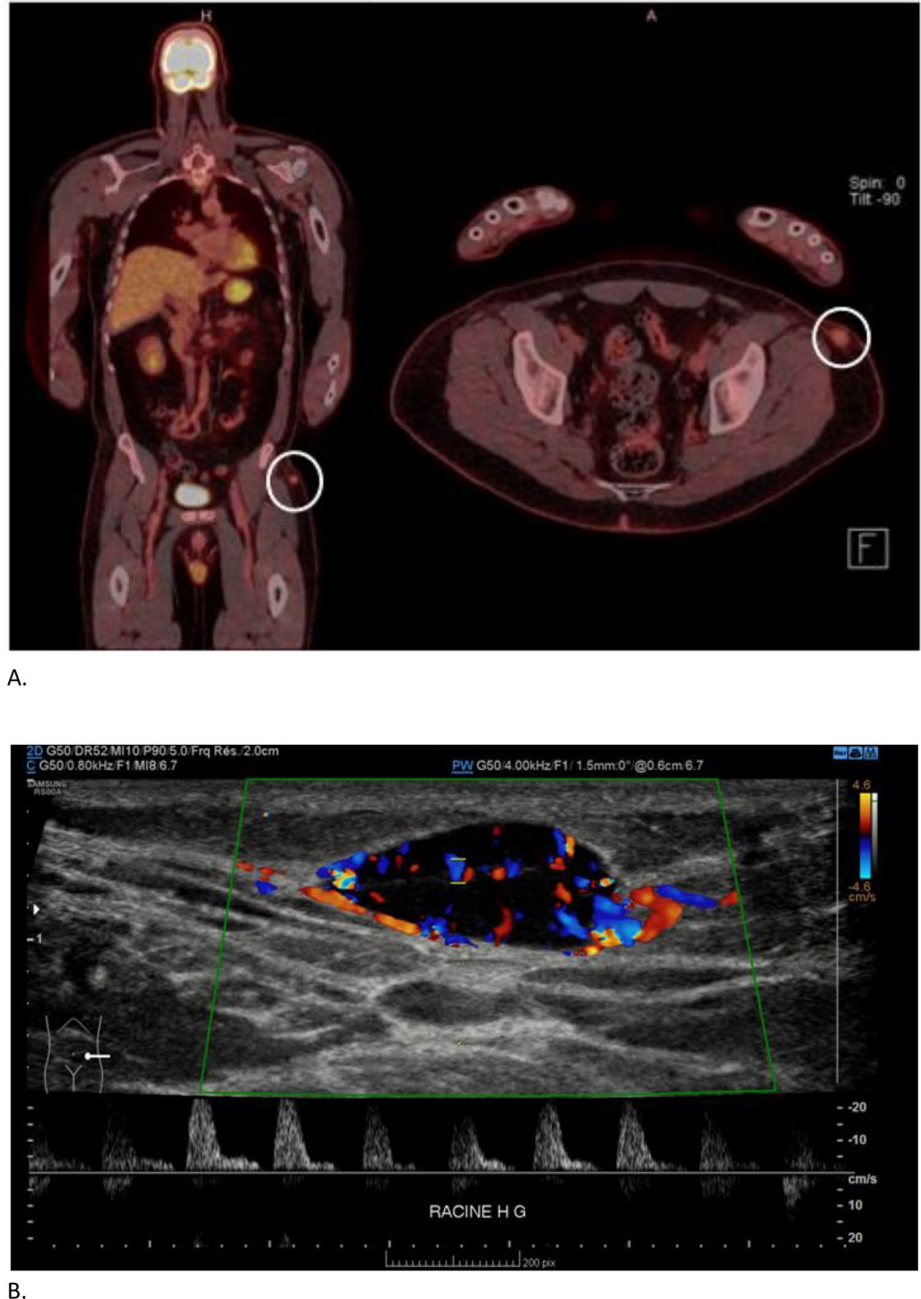
The tumor on the PET-CT and at the ultrasound scan. **A** Nodular subcutaneous hypermetabolic (max SUV 3) lesion in the left thigh. **B** Subcutaneous hypoechogenous lesion at the ultrasound measuring 17 × 8 × 14mm, vascularized with arterial and venous flow with Doppler

Due to the late onset of bone fragility, inherited causes of FS were unlikely. Ophthalmological examination did not find elements for cystinosis nor Kayser-Fleischer rings for Wilson’s disease. With the absence of hepatic or neurological involvement, and normal ceruloplasmin levels, this latter diagnosis was unlikely. A positron emission tomography and computed tomography (PET-CT 18-FDG) showed the recent vertebral and pelvic fractures, and it identified multiple recent rib fractures; moreover, it highlighted the presence of a 11 × 15 mm hypermetabolic subcutaneous lesion in the proximal left upper thigh, which was later described as an arteriovenous malformation on ultrasound (Fig. 2).

Serum levels of fibroblast growth factor-23 (FGF23) finally came back elevated at 300 pg/mL (N < 50 pg/mL). Tumor-induced ostomalacia was therefore suspected. The patient underwent surgical resection of the lesion in the left thigh. Histopathological analyses showed a benign (19 × 18 × 11mm) mesenchymatous phosphaturic tumor. Molecular diagnosis identified a fibronectin 1- fibroblast growth factor receptor 1 (FN1-FGFR1) gene fusion, encountered in over 50% of cases of phosphaturic mesenchymal tumors, therefore confirming the diagnosis.

Hypophosphatemia and other dyselectrolytemia sustainably resolved 2 days after surgery, enabling us to stop both phosphate and potassium substitution. Glycosuria also disappeared after one week. Calcium and vitamin D supplementation was continued to support a hungry bone syndrome following surgery. The patient has not had any new fracture. The DEXA scan performed 2 years after surgery showed a 37% and 16% increase in bone mineral density at the lumbar and femoral sites, respectively, with complete normalisation of Z-scores.

## Discussion

Tumor-induced osteomalacia is caused by increased levels of FGF23, usually secreted by a mesenchymal tumor. FGF23 is a phosphaturic hormone whose physiological role is to decrease phosphate levels. Usually, its production is stimulated by high levels of phosphate or 1,25(OH)_2_ vitamin D. Synthesized mainly by osteocytes and, to a lesser extent, by osteoblasts and odontocytes, FGF23 links to its receptor FGF-R and its co-receptor Klotho, which is mainly produced in the proximal renal tubule [3]. This inhibits the renal apical membrane expression of phosphate co-transporters NpT2a and NpT2c, but also of NpT1, in the proximal tubule, leading to decreased phosphate reabsorption from the kidney [4]. 1,25-(OH)_2_ vitamin D plays a key role in the production of the intestinal NpT2-b receptors, responsible for intestinal phosphate absorption from the diet [5]. FGF23 also inhibits renal 1-α hydroxylase, which subsequently leads to decreased levels of 1,25-(OH)_2_ vitamin D and diminished intestinal phosphate reabsorption [6]. In addition, FGF23 also decreases PTH synthesis. All these processes lead to hypophosphatemia when FGF23 is in excess.

The role of FGF23 was first identified in inherited conditions, such as X-linked hypophosphatemic rickets, autosomal dominant hypophosphatemic rickets and autosomal recessive hypophosphatemic rickets 1 and 2, caused by a loss-of-function of the following genes: phosphate-regulating gene with homologies to endopeptidases on the X chromosome (*PHEX*), dentine matrix protein 1 (*DMP1*) and ectonucleotide pyrophosphate/phosphodiesterase 1 (*ENPP1*), respectively [6]. Due to a defect in catabolism, FGF23, which is downregulated by *PHEX*, *DMP1* and *ENPP1*, is therefore found in excess in these pathologies [1]. Conversely, in TIO, FGF23 is usually elevated due to increased secretion [7].

Phosphaturic tumors tend to be slow-growing mesenchymal tumors which are benign in over 90–95% of cases, but malignant tumors have been described. Based on their histological characteristics, phosphaturic tumors have been subdivided into 4 groups: phosphaturic mesenchymal tumors, mixed connective tissue type-variant (PMTMCT), osteoblastoma-like tumors, ossifying fibrous-like tumors and non-ossifying fibrous-like tumors. PMTMCT is the most common subtype, occurring in 70% of cases [8]. These tumors tend to be small (median size: 2.7 cm) and can be found in any part of the body. A recent review of 895 cases of TIO by Bosman et al. found that 46.3% were localized in the lower limbs, 25.7% in the head and neck, 10.3% in the pelvis, 9.7% in the trunk and 6.7% in the upper limb [8]. Treatment relies on resection of the tumor, which leads to normalization of FGF23 and phosphate levels. Due to its varying histology and localization, finding the tumor can be challenging. These tumors tend to express receptors to somastatin, therefore ^68^ Ga-DOTATATE has proven to be the most sensitive tool to localize these tumors [8]. 18-FDG PET/CT-scan is less sensitive but detects about 67% of cases [9]. In our case, this latter highlighted the hypermetabolic lesion in the thigh, which turned out to be the tumor secreting FGF23.

Amongst other causes of raised FGF23, a genetic cause was unlikely due to the age of onset, the phenotype and the absence of suggestive family history. Our patient did not have fibrous dysplasia nor café-au-lait spots as could be found in Mc Cune-Albright syndrome, which can also associated with elevated levels of FGF23. Although chronic kidney disease is a cause of raised FGF23 [10], this latter tends to be low in FS, which led us to consider TIO with an associated FS [11]. This association is rare and tends to be seen with an incomplete FS displaying multiple electrolyte disbalances, normoglycemic glycosuria generalised aminoaciduria or hyperglycinuria alone, but often without renal failure or metabolic acidosis [12, 13]. The mechanisms by which oncogenic osteomalacia leads to FS in some patients are not fully understood. As phosphate plays a key role in energetic exchanges (ATP and adenosine diphosphate), ATP depletion may cause dysfunction of the proximal tubule, affecting the reabsorption of other electrolytes. Prolonged dysfunction of NpT2a due to raised FGF23 may also be involved. Two siblings with autosomal recessive Fanconi's syndrome and hypophosphatemic rickets were found to have a duplication of 21 bp in *SLC34A1,* which is responsible for encoding for NpT2a [14]. Interestingly, a Chinese study by Jiang et al. in which a comprehensive single-nucleotide polymorphisms screening was performed in 30 TIO patients with FS and 30 TIO patients without FS found that the polymorphisms of XPR1 and SCL34A3 were associated with FS development in TIO patients [15].

In conclusion, Fanconi syndrome associated with TIO is a rare finding, which can lead to delay in diagnosis and treatment. This case highlights the importance of measuring FGF23 levels in cases of FS of unknown origin with marked hypophosphatemia.
